# GABA Signaling in the Posterodorsal Medial Amygdala Mediates Stress-induced Suppression of LH Pulsatility in Female Mice

**DOI:** 10.1210/endocr/bqac197

**Published:** 2022-12-01

**Authors:** Caitlin McIntyre, Xiao Feng Li, Ross de Burgh, Deyana Ivanova, Geffen Lass, Kevin T O’Byrne

**Affiliations:** Department of Women and Children's Health, Faculty of Life Sciences and Medicine, King's College London, London, UK; Department of Women and Children's Health, Faculty of Life Sciences and Medicine, King's College London, London, UK; Department of Women and Children's Health, Faculty of Life Sciences and Medicine, King's College London, London, UK; Department of Women and Children's Health, Faculty of Life Sciences and Medicine, King's College London, London, UK; Department of Women and Children's Health, Faculty of Life Sciences and Medicine, King's College London, London, UK; Department of Women and Children's Health, Faculty of Life Sciences and Medicine, King's College London, London, UK

**Keywords:** LH, MePD, GABA, optogenetics

## Abstract

Psychological stress is linked to infertility by suppressing the hypothalamic GnRH pulse generator. The posterodorsal subnucleus of the medial amygdala (MePD) is an upstream regulator of GnRH pulse generator activity and displays increased neuronal activation during psychological stress. The MePD is primarily a GABAergic nucleus with a strong GABAergic projection to hypothalamic reproductive centers; however, their functional significance has not been determined. We hypothesize that MePD GABAergic signalling mediates psychological stress–induced suppression of pulsatile LH secretion. We selectively inhibited MePD GABA neurons during psychological stress in ovariectomized (OVX) Vgat-cre-tdTomato mice to determine the effect on stress-induced suppression of pulsatile LH secretion. MePD GABA neurons were virally infected with inhibitory hM4DGi-designer receptor exclusively activated by designer drugs (DREADDs) to selectively inhibit MePD GABA neurons. Furthermore, we optogenetically stimulated potential MePD GABAergic projection terminals in the hypothalamic arcuate nucleus (ARC) and determined the effect on pulsatile LH secretion. MePD GABA neurons in OVX female Vgat-cre-tdTomato mice were virally infected to express channelrhodopsin-2 and MePD GABAergic terminals in the ARC were selectively stimulated by blue light via an optic fiber implanted in the ARC. DREADD-mediated inhibition of MePD GABA neurons blocked predator odor and restraint stress-induced suppression of LH pulse frequency. Furthermore, sustained optogenetic stimulation at 10 and 20 Hz of MePD GABAergic terminals in the ARC suppressed pulsatile LH secretion. These results show for the first time that GABAergic signalling in the MePD mediates psychological stress–induced suppression of pulsatile LH secretion and suggest a functionally significant MePD GABAergic projection to the hypothalamic GnRH pulse generator.

A population of neurons coexpressing kisspeptin, neurokinin B, and dynorphin A (KNDy) in the hypothalamic arcuate nucleus (ARC) compose the GnRH pulse generator and consequently regulate pulsatile LH secretion (1, 2). Psychological stress disrupts GnRH pulse generator frequency, resulting in a suppression of reproductive function (3). The amygdala, a component of the limbic brain implicated in emotional processing, has been shown to mediate stress-related reproductive dysfunction. Whereas lesioning of the medial amygdala blocks psychological stress–induced suppression of the GnRH pulse generator (4), the underlying neural mechanisms remain to be fully established.

The posterodorsal subnuclei of the medial amygdala (MePD), has been shown to regulate the neuroendocrine response to psychogenic stress (5–7) and displays increased neuronal activation in response to various psychological stress paradigms (8–10). The MePD has also been shown to be a key upstream regulator of GnRH pulse generator frequency and pubertal timing (11–14). Intra-MePD administration of kisspeptin antagonist decreases LH pulse frequency (14), whereas selective optogenetic stimulation of MePD kisspeptin neurons increases LH pulse frequency (12). Although we have shown that MePD kisspeptin neurons do not express GABA (12), the MePD is predominantly composed of GABAergic neurons (15), which are implicated in mediating stress-related behaviors. During displays of aggression, more than 90% of activated neurons in the MePD are GABAergic (16) and GABA is upregulated in the medial amygdala of rodents exhibiting anxiety-related behaviors (17, 18). Furthermore, selective optogenetic stimulation of MePD GABAergic neurons promotes aggressive social behaviors (16, 19) whereas optogenetic inhibition of MePD GABAergic neurons terminates ongoing attack behaviors (16, 20). Moreover, GABAergic output from the medial amygdala mediates stress-induced activation of the hypothalamus-pituitary-adrenal axis (21). Approximately 80% of MePD projection neurons are GABAergic and provide efferents to key hypothalamic reproductive centers (22). Although direct projections from the MePD to ARC KNDy neurons have been identified, their neurochemical phenotype and functional significance have not yet been determined (23, 24). Considering the role of MePD GABA neurons in regulating physiological and behavioral responses to stress, MePD GABAergic signalling may also be pivotal in integrating stressful stimuli with hypothalamic GnRH pulse generator activity.

In the present study, we used chemogenetic designer receptor exclusively activated by designer drugs (DREADDs) to selectively silence MePD GABAergic neurons during psychological stress to determine the effect on GnRH pulse generator activity. To further explore the neural circuitry regulating this response, we used an optogenetic strategy to selectively stimulate potential MePD GABAergic projection neuron terminals in the ARC to determine the effect on LH pulse frequency.

## Materials and Methods

### Animals

Adult Vgat-cre mice homozygous for the allele Slc32a1tm2(cre)Lowl (Jax stock #028862, B6J.129S6(FVB)-Slc32a1^tm2(cre)lowl^/MwarJ; Jackson Laboratory, Bar Harbor, ME, USA) were cross-bred in house with adult homozygous tm9(CAG-tdTomato)Hz mice (Jax stock #007909, B6.Cg-Gt(ROSA)26Sortm9(CAG-tdTomato)Hze/J, Jackson Laboratory) to acquire double heterozygous Vgat-cre-tdtomato mice with the tomato transgene being expressed upon cre-mediated recombination in Vgat-cre-tdTomato expressing cells (25). Vgat-cre-tdTomato mice were genotyped using PCR to determine heterozygosity for Vgat-cre (primers 5′-3′: common, 12785—CTTCGTCATCGGCGGCATCTG; wild-type reverse 12786—CAGGGCGATGTGGAATAGAAA; mutant reverse oIMR8292—CCAAAAGACGGCAATATGGT) and tdtomato (primers 5′-3′: wild-type forward oIMR9020—AAGGGAGCTGCAGTGGAGTA; wild-type reverse oIMR9021—CCGAAAATCTGTGGGAAGTC; mutant reverse WPRE oIMR9103—GGCATTAAAGCAGCGTATCC; mutant forward tdTomato oIMR9105—CTGTTCCTGTACGGCATGG). Adult female Vgat-cre-tdTomato mice weighing between 19 and 21 g and aged between 6 and 8 weeks were used. All mice were group housed unless chronically implanted with a fiber optic cannula and kept under standard conditions with a 12:12 hours light-dark cycle at 25 ± 1 °C with ad libitum access to standard chow and water. The King's College London Animal Welfare and Ethical Review Body approved all animal procedures performed. Procedures were in accordance with UK home office regulation.

### Stereotaxic Surgery

All surgical procedures were carried out under general anesthesia using ketamine (Vetalar, 100 mg/kg, IP; Pfizer, Sandwich, UK) and xylazine (Rompun, 10 mg/kg, IP; Bayer, Leverkusen, Germany) under aseptic conditions. Vgat-cre-tdTomato mice were secured in a David Kopf stereotaxic frame (Kopf Instruments, Model 900) and bilaterally ovariectomized (OVX) at the time of neurosurgery. Intra-MePD bilateral stereotaxic viral injection of the inhibitory DREADD viral construct (AAV-hSyn-DIO-HA-hM4D(Gi)-IRES-mCitrine, 3 × 10^13^ GC/mL, Serotype:8; Addgene, MA, USA) was performed for the targeted expression of hM4D(Gi)-DREADD in MePD GABA neurons using a robot stereotaxic system (Neurostar, Tubingen, Germany). To reveal the skull, a midline incision was made in the scalp and 2 small holes were drilled above the location of the MePD. Coordinates for the MePD (2.30 mm lateral, −1.55 mm from bregma, at a depth of −4.94 mm below the skull surface) were obtained from the mouse brain atlas of Paxinos and Franklin (26). Either AAV-hSyn-DIO-hM4D(Gi)-mCitrine (n = 7, 200 nL, Addgene) or a control virus (n = 4, AAV-Ef1a-DIO-EYFP, 2.2 × 10^13^ GC/mL, Serotype: 9; Addgene) was bilaterally injected over 10 minutes into the MePD, using a 2-μL Hamilton microsyringe (Esslab, Essex, UK). The needle was left in position for a further 5 minutes and then removed slowly over 1 minute. Blood sampling procedures did not begin until 4 weeks after viral injection to ensure adequate DREADD and control fluorescent protein, respectively.

To determine the significance of MePD GABA projection to the ARC in influencing GnRH pulse generator activity, Vgat-cre-tdTomato (n = 6) or C57BL/6 wild-type (n = 3) mice were unilaterally injected with channelrhodopsin (ChR2) viral construct (AAV-EF1a-double floxed-hChR2(H134R)-EYFP-WPRE-HGHpA, 1.8 × 10¹³ GC/mL; Serotype:9; Addgene) into the right MePD as described previously. Two small bone screws were inserted into the skull and a small hole was drilled above the position of the ARC. A unilateral optic fiber cannula (200 µm, 0.39 NA, 1.25 mm ceramic ferrule; Thorlabs Ltd, Ely, UK) was inserted into the right ARC (0.3 mm lateral, 1.2 mm posterior to bregma and at a depth of 6.0 mm). Once in position, the optic fiber cannula was secured on the skull using dental cement (Super-Bond Universal Kit, Prestige Dental, UK) to bond to the screws and skull, and the incision of the skin was closed with suture. Procedures did not begin until 4 weeks after viral injection to ensure adequate ChR2 expression.

### Blood Sampling for LH Measurement

Following a 1-week recovery period, mice were handled daily for 3 weeks before blood sample collection to minimize handling stress during blood sampling (27). The tail tip was excised using a sterile scalpel, and mice were left to habituate in a clean cage for 1 hour. For LH measurement, 5 μL of blood was collected every 5 minutes for 2 or 2.5 hours from freely moving animals. Samples were diluted in 45 μL of 0.2% bovine serum albumin-0.05% PBS with Tween 20 (0.2% bovine serum albumin-0.05% PBS with Tween) and immediately placed on dry ice and stored at −80 °C until later analysis.

### DREADD Mediated Inhibition of GABAergic MePD Neurons During Psychological Stress Paradigms

Serial blood samples were collected for 1 hour before stress exposure to determine baseline LH pulse frequency. Thirty minutes before stress onset, mice received an IP injection of CNO (5 mg/kg, Tocris Bioscience, Bristol, UK) to selectively activate hM4D(Gi)-DREADDs and silence MePD GABA neurons. Controls received an IP injection of saline 30 minutes before stress onset. Mice infected with the control virus AAV-EYFP that did not contain the hM4D(Gi)-DREADD construct received an IP CNO injection. For predator odor exposure, 12 μL of 2,5-dihydro-24,5-trimethylthiazoline (TMT, >98%, Sigma-Aldrich) a constituent from fox urine, was pipetted into a filter paper lined Petri dish and placed centrally into the animal's cage for 1 hour. For restraint stress, mice were immobilized for 1 hour in a 50-mL falcon tube modified to allow ventilation and access to the tail for blood sampling. Blood sampling continued while mice were exposed to either predator odor exposure or restraint stress. The mice injected with the DREADDs viral construct were treated with CNO or saline in a random order, with at least 3 days, but typically 5 days, between experiments.

### In Vivo Optogenetic Stimulation of MePD GABAergic Projection Terminals in the ARC

Vgat-cre-tdTomato mice transfected with AAV-ChR2 in the MePD or wild-type controls, implanted with an optic fiber cannula in the ARC allowed for the selective photo stimulation of MePD GABAergic neuron projection terminals in the ARC. Optic fiber implants were attached to a fiber optic patch cable (Thorlabs) via a ceramic mating sleeve that allows mice to freely move while receiving blue light (473 mm wavelength) stimulation via the optic fiber implants. Laser (DPSS laser, Laserglow Technologies) intensity was set to 10 mW at the tip of the fiber optic patch cable. Frequency and pulse width were regulated by a Grass stimulator SD9B (Grass Instrument Company). After 1 hour of controlled blood sampling without optical stimulation to determine baseline LH pulse frequency, blood sampling continued while mice received 90 minutes sustained optical stimulation of MePD GABAergic projection neuron terminals in the ARC at frequencies of 2 Hz, 5 Hz, 10 Hz, or 20 Hz (10-ms pulse width) or alternatively no stimulation as a control. The Vgat-cre-tdTomato mice received all the stimulation protocols in random order, with at least 3 days between experiments. Wild-type animals received 20 Hz optic stimulation only.

### Validation of AAV Injection Site and Fiber Optic Cannula Placement

After experimentation had concluded, mice received an IP lethal injection of ketamine and were transcardially perfused with heparinized saline for 5 minutes followed by phosphate-buffered (pH 7.4) 4% paraformaldehyde for 10 minutes using a pump (Minipuls; Gilson, Dunstable, UK). Brains were placed into a 15% sucrose-4% paraformaldehyde solution. After sinking, brains were transferred into a 30% sucrose-0.2 m PBS solution and stored overnight at 4 °C. Brains were snap frozen in isopropanol on dry ice and stored at −80 °C until coronally sectioned (30 µm) using a cryostat (Bright Instrument Co., Huntingdon, UK) when every third section was collected throughout the MePD region between −1.34 and −2.70 from bregma. Sections were airdried on microscope slides and cover slipped with ProLong Anti-fade mounting medium (Molecular Probes Inc., Eugene, OR, USA). Verification of correct AAV injection site into MePD (as indicated by the expression of fluorescent protein in the MePD) and optic fiber placement in the ARC (as indicated by the position of the optic fiber tract) was performed using an Axioskop 2 plus microscope using AXIOVISION, version 4.7 (Carl Zeiss, Oberkochen, Germany). For AAV-hM4D(Gi)-mCitrine injected Vgat-cre-tdTomato mice, we determined whether MePD GABA neurons were successfully infected in the MePD region by merging td-Tomato fluorescence expressed in Vgat cells with mCitrine or EYFP fluorescence in the MePD. The number of mCitrine or EYFP positive neurons colocalized with Vgat-cre-tdTomato fluorescence in the MePD of each animal was determined using 4 sections and the average number of neurons presented is per section per MePD. The group mean percent was calculated by taking the average number of Vgat mCitrine-positive neurons out of the average number of Vgat neurons expressing tdTomato fluorescence per 4 sections and presented as mean ± SEM%. Only data from animals with both accurate AAV injection into the MePD and cannula placement in the ARC were included in the analysis. Images were taken using Axioskop 2 Plus microscope (Carl Zeiss).

### LH Pulse Detection and Analysis

Blood samples were processed using an ultra-sensitive LH ELISA as reported previously (28) using a capture antibody (monoclonal antibody, anti-bovine LHβ subunit, AB_2665514, UC Regents, CA, USA), mouse LH standard (AFP-5306A, Harbor-UCLA, CA), primary antibody (polyclonal antibody, rabbit LH antiserum, AB_2665533, Harbor-UCLA), secondary antibody (horseradish peroxidase–linked donkey anti-rabbit IgG polyclonal antibody, AB_772206, VWR International, Leicestershire, UK). Intra-assay and inter-assay variations were 3.8% and 10.7%, respectively, and the functional assay sensitivity was 0.31 ng/mL. LH pulses were determined using the DynPeak algorithm (29), with settings adjusted to accommodate for the high LH pulse frequency in OVX mice as previously outlined by Breen and colleagues (3). These include using the programs’ default parameters, except the global threshold that was increased to 35%, the nominal peak threshold was reduced to 20 minutes and the 3-point peak threshold was removed. Average LH interpulse interval (IPI) (the time between 2 LH pulse peaks) was calculated for the 1-hour control period and 1-hour stress exposure or 1.5-hour optogenetic stimulation periods. On occasions in which there were no LH pulses observed during the posttreatment period, the IPI was given a value equivalent to the posttreatment interval (60 or 90 minutes). Statistical significance was tested using a 2-way repeated measures ANOVA and post hoc Tukey test. Data were represented as mean ± SEM and *P* < 0.05 was considered significant.

## Results

### Validation of AAV Injection Site Into the MePD and Confirmation of EYFP Expressing Terminals and Correct Fiber Optic Cannula Placement in the ARC

**Figure 1.**
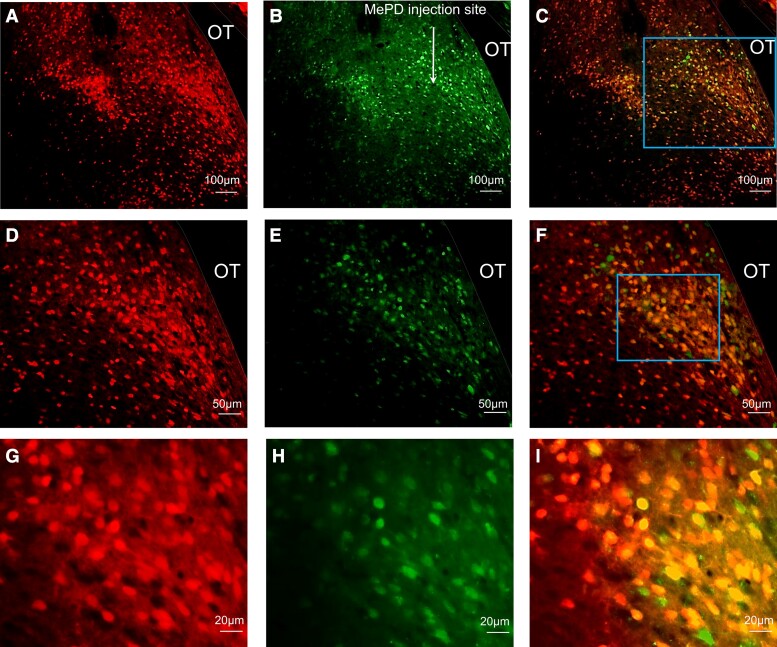
Expression of AAV-hM4D(Gi)-mCitrine in GABA neurons in the posterodorsal medial amygdala (MePD). (A-I) Representative photomicrographs of dual fluorescence in Vgat-cre-tdTomato neurons in the MePD of female OVX mice injected with AAV-hM4D(Gi)-mCitrine. Area in boxes in (C) and (F) shown at a higher magnification (D-F) and (G-I), respectively. GABA neurons labeled with TdTomato (A, D, and G), mCitrine (B, E, and H) and double labeled (C, F, and I) in the MePD. Scale bars represent (A-C) 100 μm, (D-F) 50 μm, and (G-I) 20 μm. OT, optic tract (white dashed line). White arrow (B) represents injection site of AAV-hM4D(Gi)-mCitrine into the MePD.

**Figure 2.**
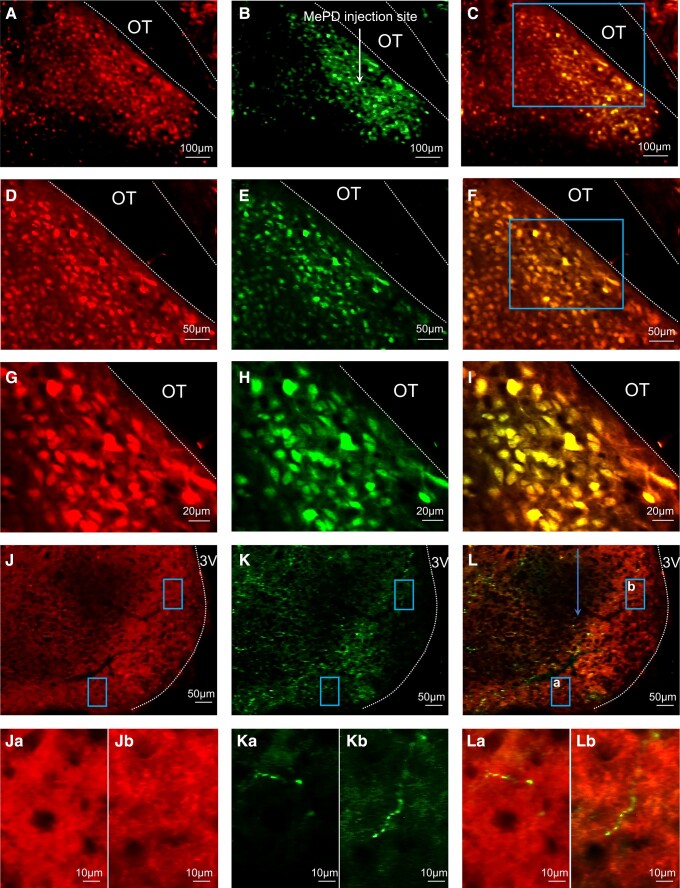
Expression of AAV-ChR2-EYFP in GABA neurons: the posterodorsal medial amygdala (MePD) and identification of MePD GABA terminals in the arcuate (ARC). (A-I) Representative photomicrographs of dual fluorescence in Vgat-cre-tdTomato neurons in the MePD of female OVX mice injected with AAV-ChR2-EYFP. Area in boxes in (C) and (F) shown at higher magnification in (D-F) and (G-I), respectively. GABA neurons labeled with TdTomato (A, D, and G), EYFP (B, E, and H) and double labeled (C, F, and I) in the MePD. OT, optic tract (white dashed line). White arrow (B) represents injection site of AAV-ChR2-eYFP into the MePD. (J-L), Representative photomicrographs of the ARC showing GABA cell bodies labeled with TdTomato (J), MePD GABA projection terminals expressing ChR2-EYFP in the ARC (K), and merged (L). (J-L a and b), Detail of areas (blue box) in (J-L), respectively, showing examples of MePD GABA terminals in the ARC (Ka, Kb, and La, Lb; scale bar 10 μm). Scale bars represent (A-C), 100 μm and (D-F) 50 μm and (G-I) 20 μm. ARC outlined with white dashed line. 3 V, third ventricle. Blue arrow in (L) represents location of fiber optic cannula.

The AAV-hM4DGi and AAV-ChR2 viruses used to infect Vgat-cre-tdTomato–positive cells in the MePD were tagged with fluorescent proteins m-Citrine and EYFP, respectively, allowing visualization under a microscope. For the chemogenetic experiments, analysis of images collected from coronal sections showed that 6 of 7 animals had successful stereotaxic injection of AAV-hM4D(Gi) virus limited to the MePD. Representative example of DREADD expression in a section of the MePD (Fig. 1A-I) showing colocalization with Vgat-TdTomato. The mean ± SEM number of mCitrine positive cells in bilaterally injected brain sections was 210.87 ± 17.49 per animal (Fig. 1B, 1E, and 1H). Evaluation of hM4D(Gi)-mCitrine expression in Vgat-cre-tdTomato neurons in the MePD show 84.55 ± 3.70% of Vgat-cre-tdTomato positive neurons coexpressed mCitrine and therefore hM4D(Gi) (Fig. 1C, 1F, and 1I). Nonspecific mCitrine expression was observed in 7.0 ± 1.2 non-tdtomato–expressing neurons in the MePD per section. For optogenetic experiments, analysis of images collected from coronal sections containing the MePD showed that 6 of 6 animals had successful infection of AAV-ChR2-EYFP virus limited to the MePD and correct placement of fiber optic cannula into the ARC. Representative example of ChR2 (Fig. 2B, 2E, and 2H) and TdTomato expression (Fig. 2A, 2D, and 2G) in a section of the MePD showing colocalization of EYFP with Vgat-TdTomato (Fig. 2C, 2F, and 2I). The mean ± SEM number of EYFP-positive cells in MePD was 220.40 ± 25.29 per animal (Fig. 2B, 2E, and 2H). Evaluation of AAV-ChR2 expression in Vgat-cre-tdTomato neurons in the MePD show 84.31 ± 8.56% of Vgat-cre-tdTomato positive neurons coexpressed eYFP and therefore ChR2 (Fig. 2C, 2F, and 2I). Nonspecific EYFP expression was observed in 7.5 ± 1.6 non-tdtomato–expressing neurons in the MePD per section. ChR2-EYFP–expressing fiber terminals were visualized predominantly in posterior sections of the ipsilateral ARC (Fig. 2K and 2L) with a density similar to previous descriptions (30). High-magnification images of ChR2-EYFP–expressing fiber terminals in the ARC are illustrated in in Fig. 2, Ka and Kb, and Fig. 2, La and Lb. The neurochemical nature of the ARC neurons that the ChR2-EYFP–expressing fiber potentially communicate with was not determined. In the wild-type mice, all 3 had correct viral injections and optic fiber placement; neither Vgat-cre-tdTomato nor EYFP was expressed.

### DREADDs Inhibition of MePD GABAergic Neurons Blocks Predator Odor Stress–induced Suppression of Pulsatile LH Secretion

**Figure 3.**
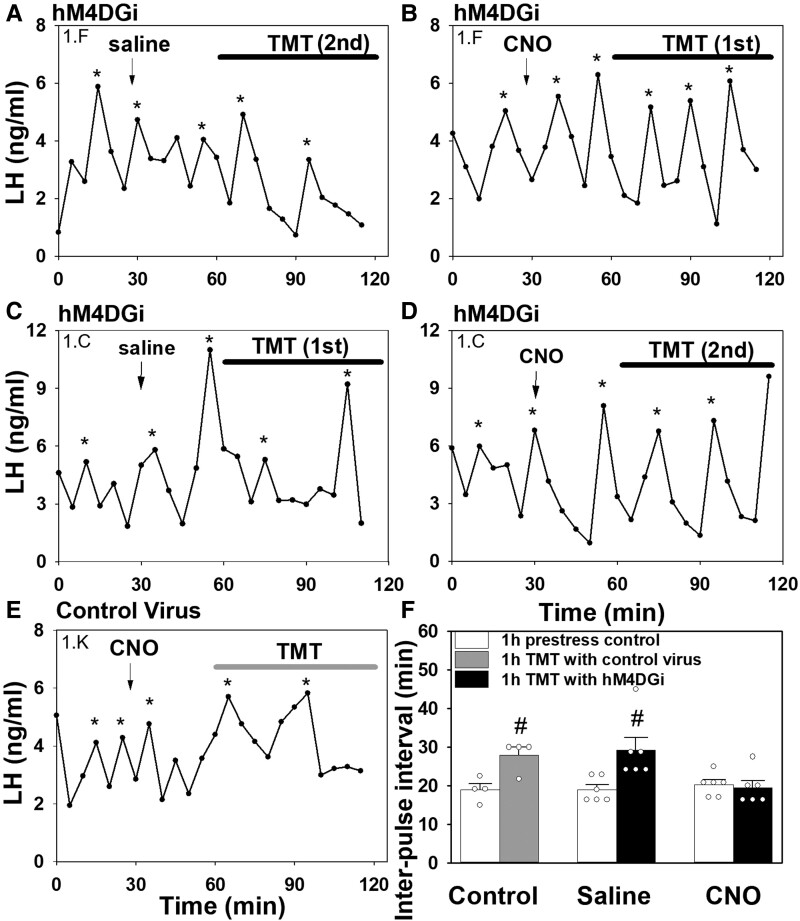
Bilateral chemogenetic silencing of GABAergic neurons in the posterodorsal medial amygdala (MePD) blocked predator odor stress–induced suppression of LH pulses in female ovariectomized Vgat-cre-tdTomato mice. Representative examples showing the effects of TMT (2,5-dihydro-2,4,5-trimethylthiazoline; 1 hour) exposure on pulsatile LH secretion in hM4D(Gi) injected mice treated 30 minutes before stress exposure with either saline (A and C) or CNO (IP 5 mg/kg) (B and D). (A) and (B) represent LH pulse profiles from the same animal (identified as 1F) exposed to TMT on the second occasion in the presence of saline treatment or the first occasion in the presence of CNO, respectively. (C) and (D) represent LH pulse profiles from the same animal (identified as 1C) exposed to TMT on the first occasion in the presence of saline treatment or the second occasion in the presence of CNO, respectively. (E) Representative example showing the effect of TMT exposure following CNO administration in control AAV-EYFP injected mice. (F) Summary of average LH interpulse interval for prestress baseline control (1 hour) and during predator odor exposure (1 hour). CNO silencing of MePD GABAergic neurons blocked the suppressive effects of predator odor exposure on LH pulse frequency. ^#^*P* < 0.05 vs prestress control period in same treatment group. Results represent the mean ± SEM, and individual data points for each animal are represented as circles in the histogram plots. LH pulses were determined using the DynPeak algorithm and are indicated with an asterisk.

Predator odor stress decreased LH pulse frequency in the saline-treated control group, increasing the mean LH IPI from 18.87 ± 1.36 minutes to 29.17 ± 3.33 minutes (mean ± SEM; n = 6 Fig. 3A, 3C, and 3F; *P* < 0.05). In contrast, bilateral inhibition of MePD GABA neurons via activation of hM4D(Gi) receptor with CNO in OVX Vgat-cre-tdTomato mice completely blocked predator odor stress–induced suppression of the LH pulse frequency compared with controls (Fig. 3B, 3D, and 3F; n = 6; *P* > 0.05). CNO administration in AAV-EYFP control virus–injected mice did not block TMT-induced suppression of pulsatile LH secretion, resulting in an increase in mean LH IPI from 18.95 ± 1.57 minutes to 27.93 ± 2.05 minutes (mean ± SEM; n = 4; Fig. 3E and 3F; *P* < 0.05). There was no significant difference between prestress baselines across groups (Fig. 3F; *P* > 0.05).

### DREADDs Inhibition of MePD GABAergic Neurons Blocks Restraint Stress–induced Suppression of Pulsatile LH Secretion

**Figure 4.**
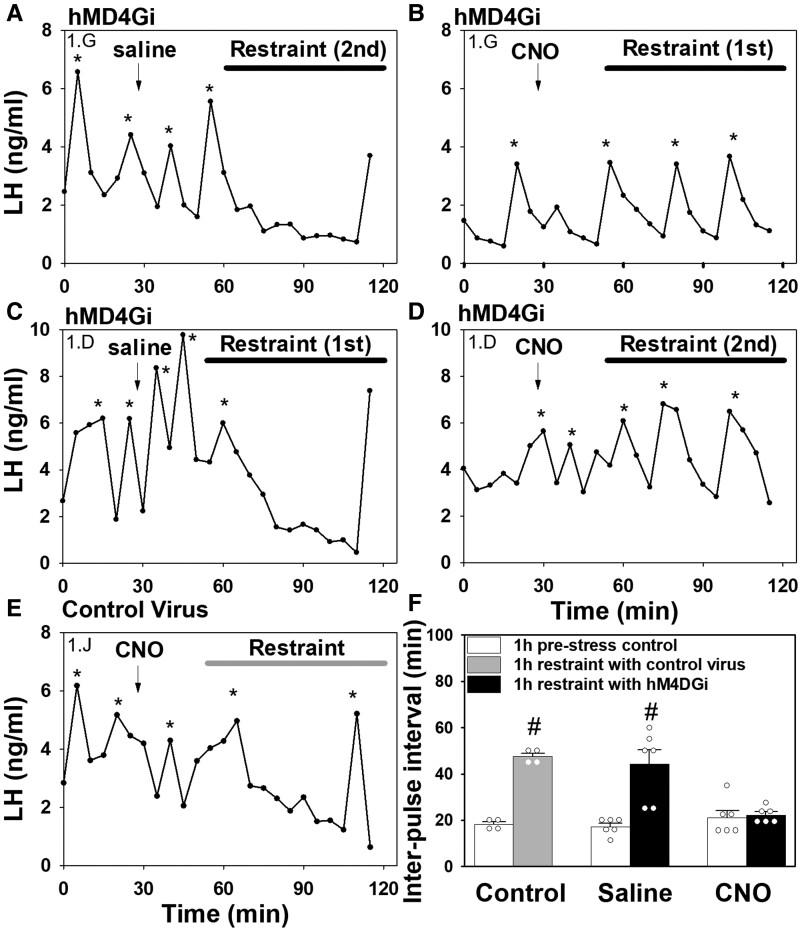
Bilateral chemogenetic silencing of GABAergic neurons in the posterodorsal medial amygdala (MePD) blocked restraint stress-induced suppression of LH pulses in female ovariectomized Vgat-cre-tdTomato mice. Representative examples showing the effects of restraint stress (1 hour) on pulsatile LH secretion in hM4D(Gi) injected mice treated 30 minutes before stress exposure with either saline (A and C) or CNO (ip, 5 mg/kg) (B and D). (A) and (B) represent LH pulse profiles from the same animal (identified as 1G) exposed to restraint stress on the second occasion in the presence of saline treatment or the first occasion in the presence of CNO, respectively. (C) and (D) represent LH pulse profiles from the same animal (identified as 1D) exposed to restraint stress on the first occasion in the presence of saline treatment or the second occasion in the presence of CNO, respectively. (E) Representative example showing the effect of restraint stress following CNO administration in control AAV-EYFP injected mice. (F) Summary of average LH interpulse interval for prestress baseline control (1 hour) and during restraint stress (1 hour). CNO silencing of MePD GABAergic neurons blocked the suppressive effects of restraint stress on LH pulse frequency. ^#^*P* < 0.05 vs prestress control period in same treatment group. Results represent the mean ± SEM, and individual data points for each animal are represented as circles in the histogram plots. LH pulses were determined using the DynPeak algorithm and are indicated with an asterisk.

Restraint stress profoundly suppressed LH pulse frequency in the saline-treated control group, dramatically increasing the average LH IPI from 17.78 ± 1.02 minutes to 44.17 ± 6.25 minutes (mean ± SEM; n = 6; Fig. 4A, 4C, and 4F; *P* < 0.001). Bilateral inhibition of MePD GABA neurons via activation of hM4Di receptor with CNO in OVX Vgat-cre-tdTomato mice completely blocked restraint stress–induced suppression of LH pulse frequency (Fig. 4B, 4D, and 4F; n = 6; *P* > 0.05). CNO administration in AAV-EYFP control virus–injected mice did not block restraint stress–induced suppression of pulsatile LH secretion resulting in an increase in mean LH IPI from 18.13 ± 1.19 minutes to 47.50 ± 1.44 minutes (mean ± SEM; n = 4; Fig. 4E and 4F, *P* < 0.05). Prestress baselines for all groups did not significantly differ (Fig. 4F; *P* > 0.05).

### Optical Stimulation of MePD GABAergic Projection Terminals in the ARC Suppresses LH Pulse Frequency

**Figure 5.**
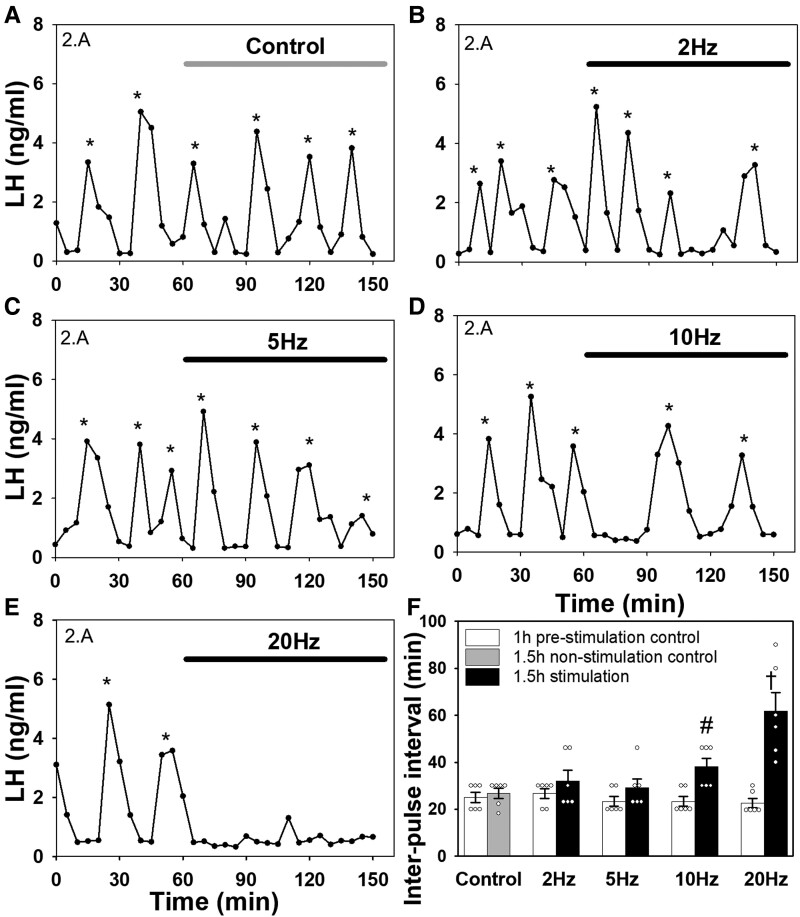
Effects of optogenetic activation of posterodorsal medial amygdala (MePD) GABAergic projection terminals in the hypothalamic arcuate nucleus (ARC) on pulsatile LH secretion. Representative examples showing the effects of no stimulation (A), 2 Hz (B), 5 Hz (C), 10 Hz (D), or 20 Hz (E) in an ovariectomized Vgat-cre-tdTomato mouse injected with an AAV-ChR2-EYFP construct in the MePD. (F) Summary of average LH interpulse interval for nonstimulation control and optical stimulation at 2 Hz, 5 Hz, 10 Hz, or 20 Hz of MePD GABAergic projection terminals in the ARC. Stimulation at 10 and 20 Hz resulted in a significant suppression of LH pulse frequency. ^#^*P* = 0.016 vs prestimulation control period for 10 Hz optical stimulation group. ^†^*P* < 0.001 vs prestimulation control period for 20 Hz optical stimulation group, and 10 Hz stimulation group. Results represent the mean ± SEM, and individual data points for each animal are represented as circles in the histogram plots. LH pulses were determined using the DynPeak algorithm and are indicated with an asterisk.

A dose-dependent suppression of LH pulse frequency was observed following optical stimulation of MePD GABA projection terminals in the ARC at 10 and 20 Hz (Fig. 5F). Selective optogenetic stimulation at 20 Hz increased the mean LH IPI from 22.64 ± 1.97 to 61.67 ± 8.02 minutes (mean ± SEM; n = 6; Fig. 5E and 5F; *P* < 0.001). Optical stimulation at 10 Hz resulted in a significant suppression of pulsatile LH secretion with an increase in mean IPI from 23.34 ± 2.11 to 38.08 ± 3.61 minutes (Fig. 5D and 5F, *P* = 0.016, n = 6). However, optical stimulation at 2 or 5 Hz, and no stimulation as a control, had no effect on LH IPI (Fig. 5A-C and 5F, *P* > 0.05, n = 6 per group). Additionally, optical stimulation in wild-type mice injected with AAV-ChR2 into the MePD and fiber optic placement in the ARC had no effect on LH IPI (22.50 ± 1.43 to 20.53 ± 2.29 minutes; mean ± SEM; n = 3; *P* > 0.05).

## Discussion

In the present study, both predator odor and restraint stress elicited a robust suppression of pulsatile LH secretion, and these effects were completely blocked by selectively silencing MePD GABA neurons using inhibitory DREADDS. MePD GABA neurons have previously been shown to regulate a range of stress-related behaviors such as aggression and infanticide. Optogenetic activation of MePD GABA neurons induces attack behaviors and conversely ongoing attack can be terminated by selective optical inhibition (16). Fiber photometric monitoring of MePD GABA neurons in mice show a dramatic increase in spontaneous activity during naturally occurring infanticidal episodes, which have been linked to stress (19, 31). During aggressive events, MePD GABA neurons display increased c-fos (16) and, similarly, the MePD demonstrates increased neuronal activity during psychological stress paradigms (6, 8). The role of MePD GABA neurons in mediating anxiogenic behaviors is consistent with our current findings that MePD GABA neurons also modulate reproductive neuroendocrine function in response to psychogenic stressors.

Discrete subpopulations of MePD GABA neurons are associated with a range of behavioral outcomes such as grooming or aggressive episodes (16); recent calcium imaging of MePD GABA neurons shows GABAergic subpopulations differentially respond to social and predator cues, suggesting behavioral outcomes are modulated by distinct subsets of MePD GABA neurons (32). Differential neuropeptide receptor expression has been shown to alter the responsiveness of MePD GABA neurons to environmental cues. Silencing oxytocin receptor expression in a subset of MePD GABAergic neurons shifts their responsiveness toward predator cues rather than social cues (33). Although the underlying neurochemical mechanism transmitting stress-related cues to MePD GABA neurons during psychological stress-induced reproductive dysfunction remains to be established, the corticotropin-releasing factor (CRF) family of stress-related neuropeptides may be a key candidate. Psychological stress activates MePD neurons expressing CRF receptor type 2 (CRF-R2) mRNA (34) and CRF-R2 expressing neurons in the medial amygdala are predominantly GABAergic (35), suggesting a CRF-R2 phenotype in MePD GABA neurons may tune subsets of neurons to stress cues rather than social ones. Intra-MePD administration of CRF delays pubertal onset and disrupts estrous cyclicity (13) and intracerebroventricular delivery of CRF-R2 antagonist blocks the suppressive effects of restraint stress on pulsatile LH secretion (36). Recently, we have shown that urocortin 3 (Ucn3) signalling, a selective ligand for CRF-R2, within the MePD is a key mediator of stress-induced suppression of the GnRH pulse generator. Administration of Ucn3 agonist into the MePD dose-dependently suppresses pulsatile LH secretion and administration of Ucn3 selective antagonist blocks the effect of psychological stressors on the GnRH pulse generator (7). Furthermore, DREADD-mediated inhibition of MePD Ucn3 neurons blocks both restraint- and TMT-induced suppression of LH pulse frequency (7). MePD GABA neurons may be coexpressing Ucn3 to suppress LH pulse frequency during psychological stress exposure or given that CRF-R2–expressing neurons are predominantly GABAergic in the medial amygdala (35), Ucn3 may be acting upstream of CRF-R2 expressing GABA neurons in the MePD to mediate stress-induced suppression of reproductive function.

Genetic fate mapping studies tracing the embryological origin of MePD inhibitory neurons using genetic markers have shown large morphological and biochemical diversity among MePD inhibitory neurons, which is generated by unique progenitor pools (37) resulting in functionally heterogenous MePD GABA subtypes (38, 39). Populations of both GABA interneurons and projection neurons with distinct electrophysiological properties have been identified in the MePD (40). However, the chemogenetic silencing approach used in the present study would have targeted both MePD GABA interneurons and projection neurons, thus disallowing functional differentiation between these 2 major populations.

Given that up to 80% of the neuronal output arising from the MePD is GABAergic, including a significant percentage of those reaching reproductive centers in the hypothalamus (15, 22, 40), we selectively interrogated MePD GABAergic projection neurons potentially reaching the ARC by optogenetically stimulating their terminals in that locus and found an optical stimulation intensity-dependent reduction in LH pulse frequency. This inhibitory effect was observed with 10- and 20-Hz optical stimulation, but not with the lower 2- and 5-Hz stimulation. It is generally considered that low frequency stimulation favors the release of neurotransmitter and high-frequency neuropeptide release (2), thus potentially tempering a GABAergic mechanism. However, GABA spillover induced by high-frequency stimulation activates postsynaptic metabotropic GABA_B_ receptors (41). Moreover, we have previously shown that intra-ARC administration of selective GABA_B_ receptor antagonist reduced restraint stress–induced suppression of LH pulses (42). It is therefore tempting to speculate that increased GABA output from the MePD to the ARC during psychological stress suppresses LH pulses through a GABA_B_ receptor–mediated mechanism.

The present study suggests a novel functional inhibitory MePD GABAergic projection capable of influencing the KNDy network underlying changes in GnRH pulse generator frequency. Recently, cre-dependent transfection of ARC kisspeptin neurons with a monosynaptic viral tracer has shown the MePD provides a direct primary afferent input to the ARC kisspeptin neurons; the GnRH pulse generator (24). This would support the hypothesis that the MePD GABAergic projection to the ARC functionally characterized in the present study may synapse directly onto ARC kisspeptin neurons. Although we histologically observed MePD GABA projection fibers in the ARC, it remains to be established which neuron phenotype they communicate with. Therefore, the caveat remains that we cannot exclude a potential indirect communication between the optically activated MePD GABAergic terminals in the ARC and the KNDy network.

Previously, we have described a population of non-GABAergic kisspeptin neurons in the MePD that modulates LH pulse frequency. Selective optogenetic stimulation of these MePD kisspeptin neurons increases LH pulse frequency (12) and conversely intra-MePD kisspeptin antagonism decreases GnRH pulse generator activity, suggesting kisspeptin is acting locally within the MePD to modulate LH pulse frequency (14). The present study confirming a functional MePD GABAergic projection to the ARC could provide a potential mechanism through which the MePD regulates LH pulse frequency in response to pheromonal and adverse stimuli. The MePD is of subpallial origin (39, 43) and recently a disinhibitory circuit between MePD kisspeptin neurons and the hypothalamic KNDy network has been proposed (12). Within this hypothetical neurocircuit, activated MePD kisspeptin neurons stimulate local GABA interneurons, which in turn inhibit MePD GABAergic projections, thereby reducing the GABAergic inhibitory tone to the ARC KNDy network, resulting in increased GnRH pulse generator frequency. Psychological stress may be altering the level of disinhibition within this circuit to increase GABAergic tone transmitted to the GnRH pulse generator, by either directly regulating the GABAergic system or by suppressing MePD kisspeptin signalling. Further work is required to test these hypothetical neural constructs.

The present studies were performed in an OVX mouse model in the absence of circulating estradiol. Estradiol replacement models have been shown to sensitize the GnRH pulse generator to the suppressive effects of CRF (44, 45), corticosterone (46), and a range of stressors in multiple species (47–50). Although we show MePD GABA signalling mediates psychological stress–induced suppression of pulsatile LH secretion in the absence of circulating sex steroids, a degree of caution should be used in applying these findings to intact or estrogen-replaced models, particularly because the MePD is highly responsive to circulating sex steroids, and a high percentage of MePD GABA cells also express estrogen receptor-α (51).

In conclusion, the present study shows that MePD GABAergic signalling is a crucial mediator of psychogenic stress–induced suppression of the GnRH pulse generator. However, it remains to be established if the suppressive effects of GABA are mediated locally within the MePD or from increased GABA output to the hypothalamic GnRH pulse generator. We show for the first time the presence of a functionally significant GABAergic projection from the MePD to the ARC, which may be a key component of the neural circuitry mediating psychological stress-induced suppression of the GnRH pulse generator.

## Data Availability

Some or all datasets generated during and/or analyzed during the current study are not publicly available but are available from the corresponding author on reasonable request.

## Abbreviations

ARC: arcuate nucleus
ChR2: channelrhodopsin
CRF: corticotropin-releasing factor
DREADD: designer receptor exclusively activated by designer drug
IPI: interpulse interval
KNDY: kisspeptin, neurokinin B, and dynorphin A
MePD: posterodorsal medial amygdala
OVX: ovariectomized
TMT: 2,5-dihydro-24,5-trimethylthiazoline
Ucn3: urocortin 3
